# Genome‐wide signatures of environmental adaptation in European aspen (*Populus tremula*) under current and future climate conditions

**DOI:** 10.1111/eva.12792

**Published:** 2019-04-02

**Authors:** Pär K. Ingvarsson, Carolina Bernhardsson

**Affiliations:** ^1^ Department of Plant Biology, Linnean Centre for Plant Biology Swedish University of Agricultural Sciences Uppsala Sweden; ^2^ Department of Ecology and Environmental Science Umeå University Umeå Sweden

**Keywords:** adaptation, climate change, gene flow, local adaptation, population structure, *Populus*

## Abstract

Future climate change has been predicted to disrupt local adaptation in many perennial plants, such as forest trees, but the magnitude and location of these effects are thus far poorly understood. Here, we assess local adaptation to current climate in European aspen (*Populus tremula*) by using environmental association analyses to identify genetic variants associated with two representative climate variables describing current day variation in temperature and precipitation. We also analysed patterns of genetic differentiation between southern and northern populations and observe that regions of high genetic differentiation are enriched for SNPs that are significantly associated with climate. Using variants associated with climate, we examined patterns of isolation by distance and environment and used spatial modelling to predict the geographic distribution of genomic variation in response to two scenarios of future climate change. We show that climate conditions at a northern reference site will correspond to climate conditions experienced by current day populations located 4–8 latitude degrees further south. By assessing the relationship between phenotypic traits and vegetative fitness, we also demonstrate that southern populations harbour genetic variation that likely would be adaptive further north under both climate change scenarios. Current day populations at the lagging edge of the distribution in Sweden can therefore serve as sources for introducing adaptive alleles onto northern populations, but the likelihood of this largely depends on naturally occurring levels of gene flow.

## 1 INTRODUCTION

How trees will respond to altered temperatures and precipitation patterns under projected changes to future climate depends largely on the genetic architecture of traits that are responsible for mediating local adaptation to current climate conditions. Earlier studies have highlighted the importance of phenology in mediating adaptation to climate (Aitken, Yeaman, Holliday, Wang, & Curtis‐McLane, 2008; Alberto et al., 2013; Savolainen, Pyhajarvi, & Knurr, 2007). Spring phenology is usually thought to have a relatively complex genetic basis, relying on temperature cues, requiring both chilling temperatures to release endodormancy and accumulated temperature sums to initiate bud break (Rohde & Bhalerao, 2007; Singh et al., 2018). Spring phenology has thus far been tracking shifts due to warmer temperatures (Menzel et al., 2006), but recent studies suggest that warmer winters and the concomitant reductions in chilling requirements may reduce the advance of spring phenology (Fu et al., 2015). Autumn phenology, on the other hand, largely relies on light cues in boreal environments (Singh, Svystun, AlDahmash, Jönsson, & Bhalerao, 2017) although interactions with temperature have been suggested for some species (Hänninen & Tanino, 2011). Since light conditions will remain constant under a changing climate, autumn phenology has been predicted to be less responsive to warmer temperatures. Studies on the effect of temperature on bud set and growth cessation in aspen are equivocal, with some studies showing that warmer temperature can delay bud set (Sivadasan et al., 2017) while other suggest that warmer temperatures may accelerate bud set, inducing even further mismatch between the annual growth cycle and length of the growing season (Kalcsits, Silim, & Tanino, 2009). The reason for these differing results can at least partly be explained by warmer temperatures having very different effects depending on when during the growth cycle they occur (Way, 2011). Compared to spring phenology, the genetic basis of bud set and growth cessation is relatively well understood and involves at least some loci with relatively large effects (Böhlenius et al., 2006; Ding & Nilsson, 2016; Wang et al., 2018), suggesting that autumn phenology traits could show more rapid adaptation in the face of climate change.

Local adaptation to large‐scale variation in climate is expected to induce phenotypic correlations with important environmental variables, such as day lengths, temperatures or precipitation (Savolainen et al., 2007). Recent studies have shown that correlations between phenotypes can also be mirrored by correlations between environment and genetic variants, where allele frequencies at loci important for local adaptation usually show large differences among populations and correlations with environment (Coop, Witonsky, Rienzo, & Pritchard, 2010). If divergent selection due to climate is strong, relative to gene flow, stable allelic clines can be established that track changes in climate variables (Lenormand, 2002; Yeaman & Otto, 2011). By correlating environmental variables with genomic data, it is thus possible to identify both environmental factors that are responsible for driving local adaptation and the genomic loci that are involved in mediating adaptation (Rellstab, Gugerli, Eckert, Hancock, & Holderegger, 2015). Once environmentally associated variants have been identified, the data can also be used to predict the fate of natural populations under a changing climate to both identify areas at risk of climate maladaptation and to predict genetic change needed to track climate change (Fitzpatrick & Keller, 2015; Supple et al., 2018).

In this study, we study how local climate is mediating adaptation in a widespread keystone species in boreal forests, European aspen (*Populus tremula*) (Bernhardsson et al., 2013; De Carvalho et al., 2010). We combine population genomic analyses with environmental associations to identify genomic regions involved in mediating local adaptation to climate in *P. tremula* populations sampled across Sweden. Genetic variants in the regions associated with climate are then to predict genomic responses to future climate change and highlight geographic regions that can be expected to suffer maladaptation. Finally, we employ published data from common garden experiments to evaluate how trees are expected to respond to altered climate conditions by assessing the relationship between key phenology traits and growth, an important fitness component in perennial plants.

## 2 MATERIALS AND METHODS

### 2.1 Sampling and genotyping

**Figure 1:**
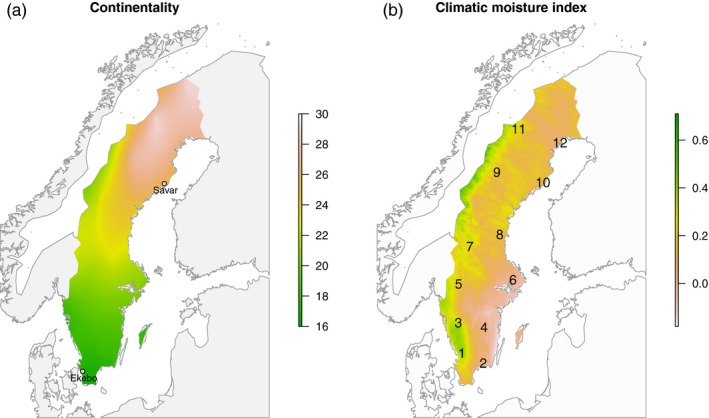
Map of variation in climate across Sweden for the two climate variables used in the analyses (a) degree days (CONT) and (b) climatic moisture index (CMI). The location of the two common garden sites is indicated with grey dots in (a), and the location of the original sample locations of the SwAsp populations (Luquez et al., 2008) is indicated with numbers in (b)

The individuals used in this study are derived from the SwAsp collection that consists of 116 unrelated *P. tremula* individuals that were from 12 sites spanning a 10° latitude degree gradient (~56–66°N) in Sweden (Figure 1, Luquez et al., 2008). In this study, we use data on 94 of these individuals that were previously genotyped by Wang et al. (2018). Briefly, DNA was extracted from all available individuals in the Swedish Aspen (SwAsp) collection (Luquez et al., 2008) and used to create paired‐end sequencing libraries with an average insert size of 650 bp. All libraries were sequenced on an Illumina HiSeq 2000 platform to a mean, per‐sample depth, of approximately 30× at the Science for Life Laboratory, Stockholm, Sweden. Raw reads were processed with Trimmomatic v0.30 (Bolger, Lohse, & Usadel, 2014) to identify reads with adapter contamination and to trim reads by removing adapter sequences and bases with quality scores lower than 20. Finally, reads shorter than 36 bases after trimming were completely discarded. All remaining reads were mapped to v1.1 of the *P. tremula* genome (Lin et al., 2018) using the BWA‐MEM algorithm with default parameters using bwa‐0.7.10 (Li, 2013). MarkDuplicates from the Picard v1.20 package (http://picard.sourceforge.net) was used to remove PCR duplicates, keeping only the read or read pair with the highest summed base quality from all pairs that had identical external coordinates. Sequencing reads in the vicinity of insertions and deletions (indels) were globally realigned using the RealignerTargetCreator and IndelRealigner in the Genome Analysis Toolkit (GATK v3.2.2) (DePristo et al., 2011). Further filtering was done to remove sites with low (less than an average of 4 × per sample) or high coverage (twice the mean depth at variant sites), sites covered by more than two reads with a mapping score of zero per sample, sites located within known repetitive sequences as identified using RepeatMasker (Tarailo‐Graovac & Chen, 2009) and sites from genomic scaffolds shorter than 2 kbp.

After filtering, genomic VCF files (g.vcf) were created for each sample using GATK HaplotypeCaller and were subsequently used to perform multi‐sample variant calling across all samples using GATK GenotypeGVCFs. SNP filtering was used to retain only high‐quality SNPs by removing SNPs at sites that did not pass previous filtering criteria and by retaining only bi‐allelic SNPs at least 5 bp away from any indels. Sites with > 30% missing data, where genotypes with quality score (GQ) lower than 10, were treated as missing, and sites showing strong deviation from Hardy–Weinberg equilibrium (*p*‐value <1e−8) were also removed. Missing SNP data were imputed, and all SNPs were phased using BEAGLE v4.1 (Browning & Browning, 2009) as described in Wang et al. (2018). Finally, SNPs were annotated using snpEff v4.3T (Cingolani et al., 2012) with a custom database based on the reference sequence and gene annotation from the v1.1 *P. tremula* draft genome (Lin et al., 2018).

### 2.2 Population genetic analyses

We used a genetic principal component analysis (PCA) to summarize variation in population structure in the SwAsp collection, using a set of putative independent SNPs that were obtained from the full data set by LD pruning using Plink v1.9. SNPs were LD pruned in windows of 20,000 SNPs with a step size of 2,000. At each step, SNPs with *r^2^* value >0.5 were pruned so that only one SNP in each pair was kept. We calculated genome‐wide levels of nucleotide diversity as well as average nucleotide diversity per population using vcftools v0.1.15 (Danecek et al., 2011).

For analyses of population structure, we divided the SwAsp individuals into three populations based on earlier results (Bernhardsson et al., 2013; De Carvalho et al., 2010; Wang et al., 2018). Individuals from populations 1 to 6 correspond to a “South” population and individuals from populations 9 to 12 to a “North.” Population 7 and 8 form a “Mid” population consisting of putatively admixed individuals (Bernhardsson et al., 2013; De Carvalho et al., 2010; Wang et al., 2018), and these were not included in the analyses of genetic differentiation. We calculated genetic differentiation between the southern and northern population using the program hapflk v1.4 (Fariello, Boitard, Naya, SanCristobal, & Servin, 2013) (downloaded from: https://forge-dga.jouy.inra.fr/projects/hapflk in September 2018). The genome‐wide hapFLK analysis was run on each chromosome separately using the following parameter values: eight clusters (−K 8), 15 EM runs to fit the LD model (−nfit=15). *p*‐Values were calculated by fitting a standard normal distribution to the genome‐wide distribution of the test statistic using R as suggested in Fariello et al. (2013).

### 2.3 Climate data

We obtained environmental data for 16 bioclimatic variables for historical (mid‐Holocene, c. 6 kya) and current (1960–1990) climate from the ENVIREM data set (Title & Bemmels, 2018) obtained at http://envirem.github.io. All analyses use ENVIREM data with a spatial resolution of 2.5 arcminute (~5 km). We also downloaded future climatic variables based on the CCSM4.0 model (http://www.cesm.ucar.edu/models/ccsm4.0/ccsm/) from Worldclim v1.4 at 2.5 arcminute resolution (Hijmans, Cameron, Parra, Jones, & Jarvis, 2005, http://worldclim.org/current). Data for future climate were obtained for the year 2070 and for two representative concentration pathways (RCP4.5 and RCP8.5), representing two future greenhouse gas concentration trajectories (Moss, Nakicenovic, & O'Neill, 2008). The Worldclim data were then used to calculate ENVIREM variables for both RCP scenarios using instructions available at http://envirem.github.io/ENVIREM_tutorial.html.

### 2.4 Environmental associations

The climate variables in the ENVIREM data set showed strong to moderate correlations within groups of variables related to temperature or rainfall. We selected two representative climate variables for use in the analyses described in this study. The first variable is the climatic moisture index (CLI), a metric of relative wetness and aridity. The second variable used is continentality (CONT) which measures the difference between the average temperature of warmest month and the average temperature of the coldest month. These variables were selected to represent climate variation related to precipitation (CLI) and temperature (CONT). We obtained data for these variables from the original sampling location of all SwAsp individuals for all time points and climate scenarios using the envirem package in R. CMI and CONT were only weakly correlated (*r *= −0.0822) across sampling locations, with CMI varying mainly along an east to west gradient, whereas variation in CONT was largely arranged along a south to north gradient (Figure 1).

The two climate variables were subsequently analysed using latent factor mixed models (LFMMs) to test for associations with genetic variants, as implemented in the R package lfmm (Frichot, Schoville, Bouchard, & François, 2013). LFMMs are a computationally efficient statistical regression models that can be used to test for associations between a multidimensional set of response variables and a set of variables of interest. The response variable in this case is individual genotypes, and the explanatory variables are environmental variables at the site of origin. LFMMs include so‐called “latent factors” which are unobserved variables that correct the model for confounding effects due to hidden factors, such as population structure. The number of latent factors to include in the model (*K*) was varied from *K* = 2 to *K* = 6, and the results were compared to assess how well the latent factors were able to control for effects of population structure and other hidden causes. All SNPs showing significant association with either of the two climate variables at a nominal *p*‐value of 1 × 10^‐6^ were extracted and used for general dissimilarity modelling analyses (see below). We also estimated the overlap between the location of significant SNPs from the LFMM analyses and outliers identified in the hapFLK analysis described in the preceding section, to determine whether genomic regions showing strong genetic differentiation between populations also are enriched for SNPs associated with environmental variation.

### 2.5 General dissimilarity modelling

We assessed the importance of the two environmental variables in explaining genetic differentiation between populations across the latitudinal gradient using generalized dissimilarity modelling (GDM; Fitzpatrick & Keller, 2015), which employ matrix regression to estimate nonlinear relationships between genetic and environmental distances (Ferrier, Manion, Elith, & Richardson, 2007).

Geographic distance between populations was calculated from GPS coordinates of the original sampling locations using the function earth.dist from the R package fossil v0.3.7 (Vavrek, 2011). Pairwise genetic differentiation was calculated based on 100,000 SNPs randomly selected from the LD pruned set of SNPs and are hereafter referred to as “reference SNPs.” We also calculated pairwise genetic differentiation from SNPs significantly associated with either of the two climate variables from the LFMM analyses. Since many of the associated SNPs showed evidence for high levels of linkage disequilibrium (LD), we pruned a set of 1,080 climate‐associated SNPs using LD clumping with Plink v1.9. LD clumping was run based on the *p*‐values obtained from the LFMM analyses, using a *p*‐value of 10^−6^ for selecting index SNPs, a *p*‐value of 10^−3^ for secondary SNPs, an *r^2^* value of 0.5 and a maximum distance of 50 kb as thresholds for clumping SNPs. After LD clumping, 111 putatively independent genomic regions associated with climate variation remain and will hereafter be referred to as “associated SNPs.” Genetic and geographic distances were compared for both “reference” and “associated” SNPs using Mantel tests calculated with the vegan R package (v2.4–2).

We estimated separate GDMs for the reference SNP set and associated SNPs, respectively. Genetic distances between populations, from the pairwise *F*_ST_ matrix, were scaled to lie between 0 and 1 by subtracting the minimum value and then dividing by the maximum value (Fitzpatrick & Keller, 2015). Scaling was performed to enable comparisons between reference and associated SNPs that displayed different ranges of observed *F*_ST_ values (−0.0024 to 0.0053 for reference SNPs and −0.034 to 0.195 for associated SNPs). We generated GDM models using the gdm function from the gdm package in R (v1.3.11, Manion et al., 2018) using genetic distance matrices, geographic distances between sampling sites and environmental distances calculated from the two bioclimatic variables (CMI and CONT). The results from the GDM analyses were used to predict the genetic change (genetic offset sensu Fitzpatrick & Keller, 2015) needed to track a changing climate relative to future climate conditions under the RCP4.5 and RCP8.5 scenarios (Moss et al., 2008). We also evaluated the similarity of climate conditions at the northern common garden site under the two RCP scenarios to current day climate. To compare future and current day climate, we calculated the Mahalanobis distance (*D_M_*) between future climate at the northern common garden site and current climate across Sweden. Climate similarity was calculated by subtracting the maximum *D_M_* value and scaling the resulting values to between 0 and 1.

### 2.6 Estimates of phenotypic selection and fitness landscapes

We obtained data on growth, bud flush and bud set from two common garden collections of the SwAsp individuals located at Ekebo (55.9°N) and Sävar (63.4°N) (Hall et al., 2007; Luquez et al., 2008; Michelson et al., 2018). Measuring fitness in long‐lived perennial trees is extremely hard, and we have resorted to using growth rates as a proxy for fitness. We collected data on height and diameter from both common gardens in 2008 and calculated relative growth rates (RGR) using data on initial sizes at the time of planting in 2004. RGR data were then used to calculate relative fitness of all individuals separately for the two common garden sites at Ekebo and Sävar by scaling RGR values to between 0 and 1. We also obtained data on bud flush and bud set, two phenology traits that are important for climate adaptation in aspen (Hall et al., 2007; Luquez et al., 2008; Michelson et al., 2018). Breeding values for bud flush and bud set, calculated from clonally replicated individuals in the two common gardens, were obtained from earlier publications (Hall et al., 2007; Luquez et al., 2008; Michelson et al., 2018).

To estimate natural selection acting on phenology, we employed the multiple regression approach of Lande and Arnold (1983) to obtain estimates of linear selection coefficients gradients (β), measuring directional selection acting on the traits, or quadratic selection gradients (γ), measuring stabilizing, disruptive or correlational selection (see for a more extensive discussion, Brodie, Moore, & Janzen, 1995). Prior to analysis, phenotypic traits were standardized by subtracting the mean and dividing by the standard deviation. For analyses of selection gradients, we employed the method developed by Morrissey and Sakrejda (2013) that explicitly model non‐normal distributions of fitness as implemented in the R package gsg (Morrissey & Sakrejda, 2014). To determine the significance of the selection gradients, we used 1,000 bootstrap replicates. We also visualized the relationship between trait values and fitness using methods outlined in Morrissey and Sakrejda (2013, 2014).

## 3 RESULTS

We obtained sequencing data for 94 of the 116 original individuals from the SwAsp collection. The number of individuals genotyped per population ranged from 4 (population 11) to 10 (population 10) with a median number of 6. After SNP calling and filtering, a total of 8,007,303 SNPs remained for downstream analysis. For the LFMM analyses, we filtered the data set on minor allele frequency (MAF), keeping only SNPs where the MAF exceeded 0.05, resulting in a data set of 4,404,968 SNPs.

### 3.1 Genetic diversity and population structure

**Figure 2:**
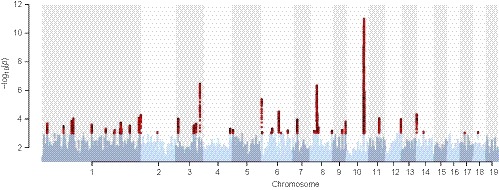
Manhattan plot of the results from the hapFLK analysis between the southern (pop 1–6) and northern (pop 9–12) populations in the SwAsp collection. Highlighted points (red) represent significant outliers (*p* < 0.001)

Nucleotide diversity was similar across the 12 populations (mean π 0.00325, range 0.00321–0.0034) and showed no relationship with geographic location. Population differentiation across the entire range of *P. tremula* in Sweden is very low (overall *F*_ST_ = 0.0021, Wang et al., 2018), and there is only a weak pattern of geographic structure in the PCA plot (Supporting Information Figure S1). Despite the low overall population differentiation seen in the SwAsp population, we do observe significant variation in genetic differentiation across the genome with several regions showing substantially elevated levels of genetic differentiation both when estimated using hapFLK (Figure 2) or when estimated using *F*_ST_ (Supporting Information Figure S2). In total, 11,055 SNPs were significant in the genome‐wide hapFLK scan, representing 35 independent genomic regions (Figure 2).

### 3.2 Environmental associations at individual loci and calculation of polygenic scores

**Figure 3:**
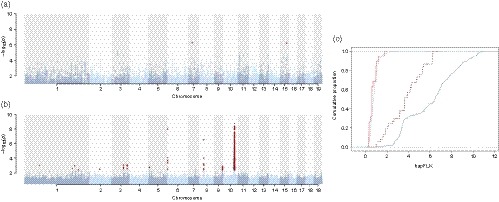
Results from the LFMM analyses. SNPs associated with the two climate variables (a) climatic moisture index (CMI) and (b) continentality (CONT) at *p* < 1 × 10^−6^ are highlighted in red. Note the different scales on the *y*‐axis in a and b. (c) Empirical cumulative distributions of hapFLK values for all SNPs (light blue) and for the SNPs that are significantly associated with CMI (light red). For CONT, the two curves represent all significant SNPs (dark blue) or SNPs excluding the region on chr10 (dashed dark red). Both curves are significantly different from the genome‐wide curve (light blue) (Kolmogorov–Smirnov test, *p* < 2 × 10^−16^ for both comparisons)

Analyses with varying values of *K* indicated that *K* = 2 adequately controlled for potentially confounding effects of population structure in the LFMM analyses (Supporting Information Figure S3). Using LFMM, we identified 19 and 1,061 SNPs that were significantly associated with climatic moisture index (CMI) and continentality (CONT), respectively (Figure 3). There was no overlap between SNPs associated with CMI and CONT. The large difference in number of associated SNPs between the two climate traits is largely explained by a region on chromosome 10 that harbour 1,024 out of the 1,061 SNPs that are associated with CONT. This region, encompassing c. 700 kb, has previously been shown to be the result of a recent selective sweep centred on the *PtFT*2 gene (Wang et al., 2018). There is a significant enrichment of SNPs that are also outliers in the hapFLK analyses among the environmentally associated SNPs, as 967 of the 1,080 environmentally associated SNPs are also among the 11,055 SNPs that have significant hapFLK values (Figure 3c) and this enrichment remains even when the large region on chr 10 is excluded (Figure 3c). Even though the majority of climate‐associated SNPs identified are noncoding (946 out of 1,080), almost all associated SNPs (1,057) are located in the vicinity of genes (i.e., within 5 kb upstream or downstream of a gene).

### 3.3 Genetic changes in response to future climate

**Figure 4:**
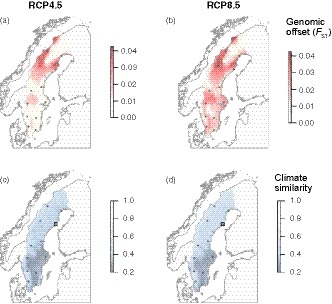
Mean genetic offset for two future climate scenarios (a) RCP4.5 and (b) RCP8.5 in 2070. Map units are in *F*_ST_ relative to current day populations. Darker red means greater genetic change needed to track a changing climate. Climate similarity between the northern common garden (location marked by a square) in 2070 and current day climate for (c) RCP4.5 and (d) RCP 8.5. Small circles mark the original SwAsp sampling locations depicted in Figure 1

To study the genomic composition of *P. tremula *across Sweden and how this relates to variation in current and future climate, we used general dissimilarity modelling (GDM) to model the relationship between genetic differentiation and geographic and environmental distance. We used a set of 100,000 SNPs, randomly sampled from across the *P. tremula* genome, to use as a “reference” set of genetic variants that are not related to climate variation. We then compared results from the “reference” data set consisting of 111 SNPs selected to be representative of the 1,080 SNPs that were climate‐associated in the LFMM analyses, as described above. Using a Mantel test, we observe significant isolation by distance for both “reference” and “associated” SNPs, but the relationship is substantially stronger for the “associated” SNPs (*r* = 0.608 and *r* = 0.894, respectively, Supporting Information Figure S4). The GDM model for the “reference” SNP set explain 40.9% of the variation in genetic differentiation (pairwise *F*_ST_) among the SwAsp populations, whereas the “associated” SNP set explain a substantially larger fraction, 84.3%, of the variation in pairwise *F*_ST_ (Supporting Information Figure S5). To project the final GDM model onto current and future environmental conditions, we delineated our model to the Swedish distribution range of *P. tremula*. The GDM model based on the “associated” SNPs was projected onto future climate conditions using two representative concentration pathway scenarios (RCP4.5 and RCP8.5, Moss et al., 2008), resulting in an estimate of genetic differentiation between current day populations and those adapted to the 2070 climate (i.e., “genetic offsets” sensu Fitzpatrick & Keller, 2015). This provides a way to quantify the amount of genomic change required to keep pace with a changing climate. These results (Figure 4) highlight the relatively large genetic change needed in the northern populations (populations 9–12) to track a changing climate. The southern populations (population 1–6) are predicted to require only comparably small genetic changes over the corresponding time period.

As a way to further visualize climate change, we evaluated the future climate at the northern common garden site under the two RCP scenarios (Figure 4c,d) with respect to current day clime. The expected climate at the northern common garden site in 2070 is most similar to that experienced by current day populations 2, 4 and 6 (Figure 1).

### 3.4 Phenotypic selection and fitness landscapes

**Figure 5:**
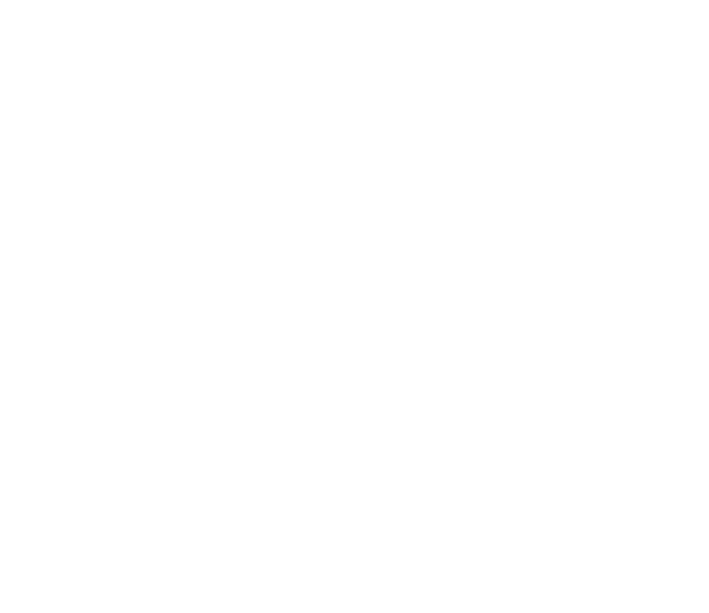
Fitness functions for bud set at (a) Ekebo and (b) Sävar and for bud flush at (c) Ekebo and (d) Sävar. Individuals from the northern populations (pop 9–12) are coloured in light blue, and individual from the southern populations (pop 1–6) are coloured in light red. The x‐axis in all plots denotes standardized trait values, and the y‐axis denotes relative fitness

To assess how variation in two putative adaptive phenology traits, bud flush and bud set, is related to vegetative fitness (growth rates), we estimated linear and quadratic selection gradients using data from both common gardens. The results from the selection analyses are summarized in Table 1, and corresponding fitness landscapes are visualized in Figure 5. There is strong selection favouring delayed bud set in both common gardens. For bud flush, selection favours trees that flush early in both Ekebo and Sävar although the magnitude of selection is substantially weaker compared to bud set. None of the traits experience quadratic (stabilizing, disruptive or correlational) selection as all γ coefficients are small and nonsignificant (Table 1).

**Table 1:** Estimated standardized selection gradients for bud set and bud flush from the Ekebo (south) and Sävar (north) common gardens

Site	Coefficient	Estimate	*SE*	*p*‐value
Ekebo	β_bud flush_	−0.127	0.036	0.002
	β_bud set_	0.395	0.036	0.000
	γ_bud flush_	0.000	0.000	0.802
	γ_bud set_	−0.007	0.051	0.958
	γ_bud set × bud flush_	−0.060	0.034	0.070
Sävar	β_bud flush_	−0.116	0.050	0.040
	β_bud set_	0.345	0.045	0.000
	γ_bud flush_	−0.095	0.060	0.330
	γ_bud set_	−0.056	0.044	0.354
	γ_bud set × bud flush_	0.028	0.042	0.566

Note

Standard errors and *p*‐values are calculated based on 500 bootstrapping replicates.

## 4 DISCUSSION

The Swedish population of *P. tremula* displays overall low levels of population differentiation (*F*_ST_ = 0.0021), despite that fact that the population included in the study cover more than 10 latitude degrees (Figure 1). The population differentiation estimated from the SNP data is substantially lower than what we have previously estimated using either SSRs (Hall et al., 2007) or SNPs (Ma, Hall, Onge, Jansson, & Ingvarsson, 2010). A number of reasons can help explain this. Genetic markers in the earlier studies were ascertained using very different criteria compared to the whole‐genome re‐sequencing employed here, making it hard to directly compare results. Also, averaging *F*_ST_ values across loci is known to bias the mean *F*_ST_ value downwards when a large number of rare variants are included (Bhatia, Patterson, Sankararaman, & Price, 2013). With the 4.4 M SNPs we use in the current study, such bias can be appreciable and thus likely contribute to the overall very low estimates of *F*_ST_ we observe in the genome‐wide data. In fact, in both the PCA and the LFMM analyses, the first axis explains around 1.3% of the variation in the SNP data (Supporting Information Figures S1 and S3) and this estimate is more in line with *F*_ST_ estimates from both Hall et al. (2007) and Ma et al. (2010), suggesting that our genome‐wide *F*_ST_ value might be downwardly biased and hence underestimate true population differentiation in *P. tremula*.

The low levels of population differentiation seen in the Swedish populations of *P. tremula* suggest that gene flow among local populations is sufficient to almost eliminate genetic differentiation despite large geographic distances separating populations (>1,000 km) for the most distant pair of populations (De Carvalho et al., 2010; Wang et al., 2018). Despite the overall low levels of genetic differentiation among Swedish *P. tremula* populations, we nevertheless identify several genomic regions that show very strong genetic differentiation between the southern (1–6) and northern (9–12) populations. We analysed genetic differentiation using the hapFLK statistic which rely on differences in haplotype frequencies between populations (Fariello et al., 2013) and using haplotype information has earlier been shown to improve the power to detect population differences driven by natural selection, compared to more traditional *F*_ST_‐based analyses based on allele frequencies at individual genetic variants. We also detected a relatively large number of SNPs that were significantly associated with climate from the LFMM analyses, with the majority being associated with CONT. This is perhaps expected since climate variation across Sweden is largely arranged along latitude and which is correlated with CONT. The absolute majority of climate‐associated SNPs are preferentially associated with genic regions (±5 kb from genes) implying that many of these SNPs likely have functional consequences. Finally, we observe that genomic regions showing enhanced genetic differentiation are also strongly enriched for SNPs that are significantly associated with CONT (Figure 3c), implying that many of these regions have diverged due to climate‐driven natural selection. It is worth noting that the enrichment remains regardless of whether the large block of SNPs located in the highly significant region on chr10 are included or not (Figure 3c). There is no overlap between regions showing strong genetic differentiation and associations to CMI but this should come as no surprise, since CMI mainly vary along longitude (Figure 1) whereas the genetic differentiation analyses compare populations along latitude. The climate‐associated SNPs collectively also show substantially stronger isolation by distance than the genome‐wide background, with pairwise *F*_ST_ values among populations that are up to 30 times higher than the genome‐wide average.

Both the hapFLK scan and the LFMM analyses recover a very strong signal on chr 10 containing c. 95% of all climate‐associated SNPs. This region is centred on the *PtFT2 *locus, a gene we have earlier shown to be involved in mediating local adaptation in phenology and climate (Wang et al., 2018). While the region on chr 10 displays the strongest signal of climate adaptation, there are a number of other genome regions that also show evidence for local adaptation through significant associations with climate and strong genetic differentiation. Regions showing both high genetic differentiation and SNPs associated with climate variables can be found on chromosomes 3, 5, 8 and 9 and they harbour a number of potentially interesting candidate genes. Among the genes implicated in climate adaptation from the LFMM analyses are two senescence‐associated genes (*Potra008949g2626*1 and *Potra002821g20059*). Leaf senescence also shows clinal variation in the SwAsp populations, although it does not seem to be directly triggered by photoperiod (Michelson et al., 2018). The LFMM analyses also identify several genes involved in growth and development of roots and leaves. The peak on chr8 for CONT houses a laccase gene putatively involved in root elongation in response to dehydration (*Potra001886g15018*). Similarly, *P. tremula* homologs of *BG*1 (*Potra002139g1657*0), *GFR*9 (*Potra000632g04828*) and *ARF*8 (*Potra001409g11957*) are associated with CMI and are all involved in mediating growth and root or leaf development and are intriguing candidates for adaptation to variation in precipitation. More information on the putative candidate genes can be found in Supporting Information Table S1. All these genes are clearly worth further studies to evaluate whether and how they may be involved in mediating adaptation to climate in *P. tremula*.

The effects of projected climate change will not affect current day populations equally, with genetic offsets substantially larger in northern Sweden under both RCP scenarios (Figure 4). The effects of climate change predicted for the northern populations are also illustrated by a comparison of the future climate at the northern common garden with current day conditions (Figure 4c,d). By 2070, the climate at the northern common garden is expected to be most similar to current day populations that are located 4–8 latitude degrees further to the south. Similar results have been observed in *Populus balsamifera*, where Keller, Chhatre, and Fitzpatrick (2017) suggested more severe effects of climate change in leading edge compared to lagging edge population.

Despite the large genetic change predicted in northern populations, patterns of genetic variation in key phenology traits, such as bud flush and bud set, in the common gardens suggest that natural selection could allow populations to track a changing climate (Figure 5). Trees with extended growth seasons, that is, trees with early bud flush and late bud set, show higher growth rates, although the results are generally weaker for bud flush (Table 1, Figure 5). Trees from southern populations are showing superior growth in the northern common garden (Figure 5), suggesting that if gene flow can introduce sufficient genetic variation natural selection with populations may, at least partly, be able to track a changing climate. To what extent this is possible depends on how effective gene flow is in introducing variation that will be adaptive under future climate conditions. If gene flow is as high as the low population differentiation we observe in *P. tremula* appears to indicate, it is possible that this could be achieved without the need for management interventions such as assisted migration (Aitken & Bemmels, 2016). However, these analyses critically rely on the assumptions that we can accurately judge rates of gene flow from current levels of population differentiation, as discussed above, and that growth is a good proxy for assessing fitness in *P. tremula*. Measuring fitness in perennial plants is fraught with difficulties, but a common rationale for using growth rate as a proxy for vegetative fitness is that early mortality, following seedling establishment, is high in most forest trees and individuals that grow rapidly will often tend to outcompete neighbours (Collet & Le Moguedec, 2007; Peet & Christensen, 1987). Furthermore, tree crown volume of adult trees, and hence potential flower production, is highly correlated with basal area and therefore also growth rate (Bush, Smouse, & Ledig, 1987; Chisman & Schumacher, 1940).

Forest management has traditionally relied on extensive provenance trials to assess response to climate variation and to identify suitable seed sourcing locations. However, a number of recent studies (Martins et al., 2018; Rellstab et al., 2016; Supple et al., 2018) have shown that combining genomic information with climate modelling provides a novel and potentially faster way forward for assessing the climate change‐induced risks. This approach allows for predictive modelling of the possible consequences of climate change and should help identify areas and/or populations where management interventions are needed to ensure persistence (Fitzpatrick & Keller, 2015; Supple et al., 2018). In this study, we have integrated information on variation in key climate variables with phenotypic variation measured at multiple common garden sites and information on whole‐genome variation in a keystone deciduous tree species, *P. tremula* (Bernhardsson et al., 2013; De Carvalho et al., 2010). We identify a number of genomic regions and putative candidate genes that are strongly associated with variation in climate and use this information to predict the ability of population to evolve responses to future climate change. Our results show that while the expected genetic changes are not overly large, they will affect populations across the latitudinal gradient differently. Leading edge populations are expected to experience greater genetic change in order to track a changing climate compared to lagging edge populations. Common garden data suggest that if appropriate genetic variation can be introduced into these populations, either through natural means or by assisted migration, natural selection could possibly allow the populations to track future environmental change. In conclusion, our study presents compelling evidence that significant portions of the distribution range of *P. tremula* in Scandinavia will experience drastically changing climates in the not so distant future and that this will induce strong selection on local populations. Whether the species can adapt to a changing climate over these timescales will largely depend on the availability of sufficient adaptive variation within local populations which in turn depends on how rapidly gene flow can introduce such variation.

## DATA ARCHIVING STATEMENT

All sequence data used in this paper are already publicly available. All raw sequencing reads have been deposited in NCBI's Sequence Read Archive (SRA) under accession number PRJNA297202 (https://www.ncbi.nlm.nih.gov/bioproject/PRJNA297202/). Filtered variant calls in VCF format are available for download from ftp://plantgenie.org/Data/PopGenIE/Populus_tremula/v1.1/VCF/. Phenotypic data are available through Zenodo (http://zenodo.org) with https://doi.org/10.5281/zenodo.2454463 under a CC BY‐SA 4.0 license. Scripts to reproduce the analyses are available from GitHub https://github.com/parkingvarsson/PopulusClimateAdaptation under a MIT License.

## CONFLICT OF INTEREST

None declared.
