# Supplementary material for: Genome‐wide signatures of environmental adaptation in European aspen (Populus tremula) under current and future climate conditions
Source: Evol Appl. 2019 Apr 2;13(1):132–42. doi: 10.1111/eva.12792 (PMC6935590; doi:10.1111/eva.12792)
Supplement: Supplementary file 1 [file EVA-13-132-s001.pdf]

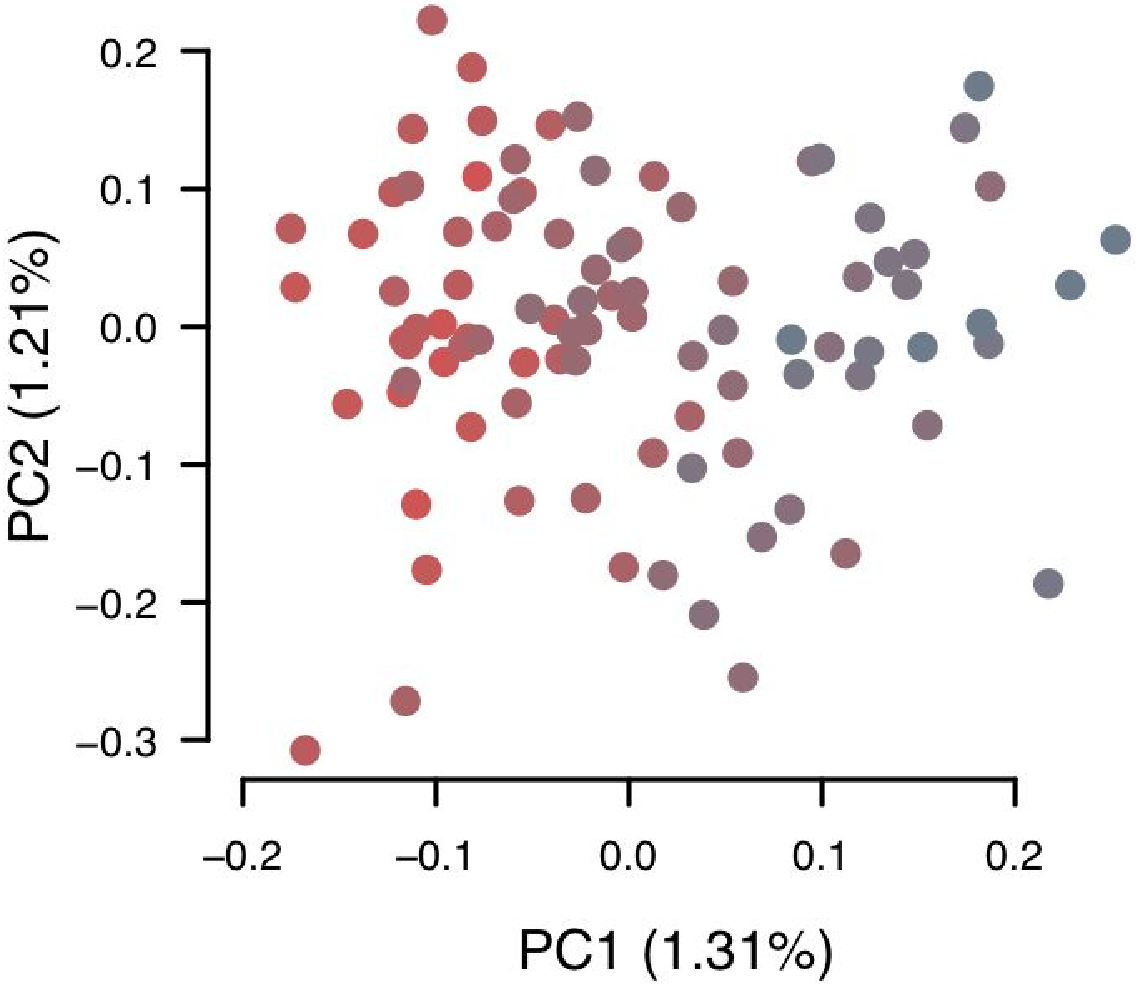


**Supplementary Figure 1.** Principal component analysis of population structure in the SwAsp collection based on a PCA of 760,292 SNPs that have been pruned to remove SNPs in high linkage disequilibrium and with a minor allele frequency exceeding 0.05. Although two PCA axes are shown, only the first axis display significant population structure (1.31% variance explained). Samples are colored according to location, ranging from south (red) to north (blue). Modified from Wang et al. (2018).

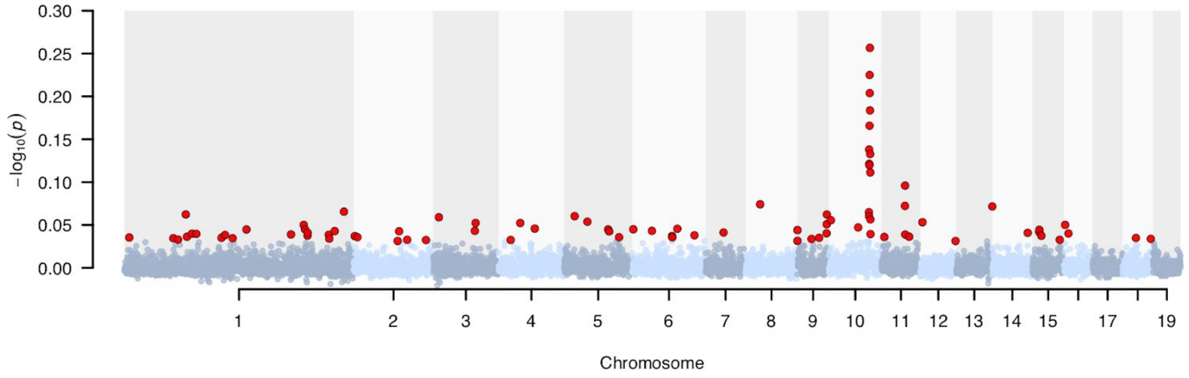


**Supplementary Figure 2.** Manhattan plot of genetic differentiation between the southern and northern populations in the SwAsp collection, calculated in 50kb sliding windows (increment 20kb) across the *P. tremula* genome. Highlighted windows (red) are significant at a genome-wide level of $p<0.001$.

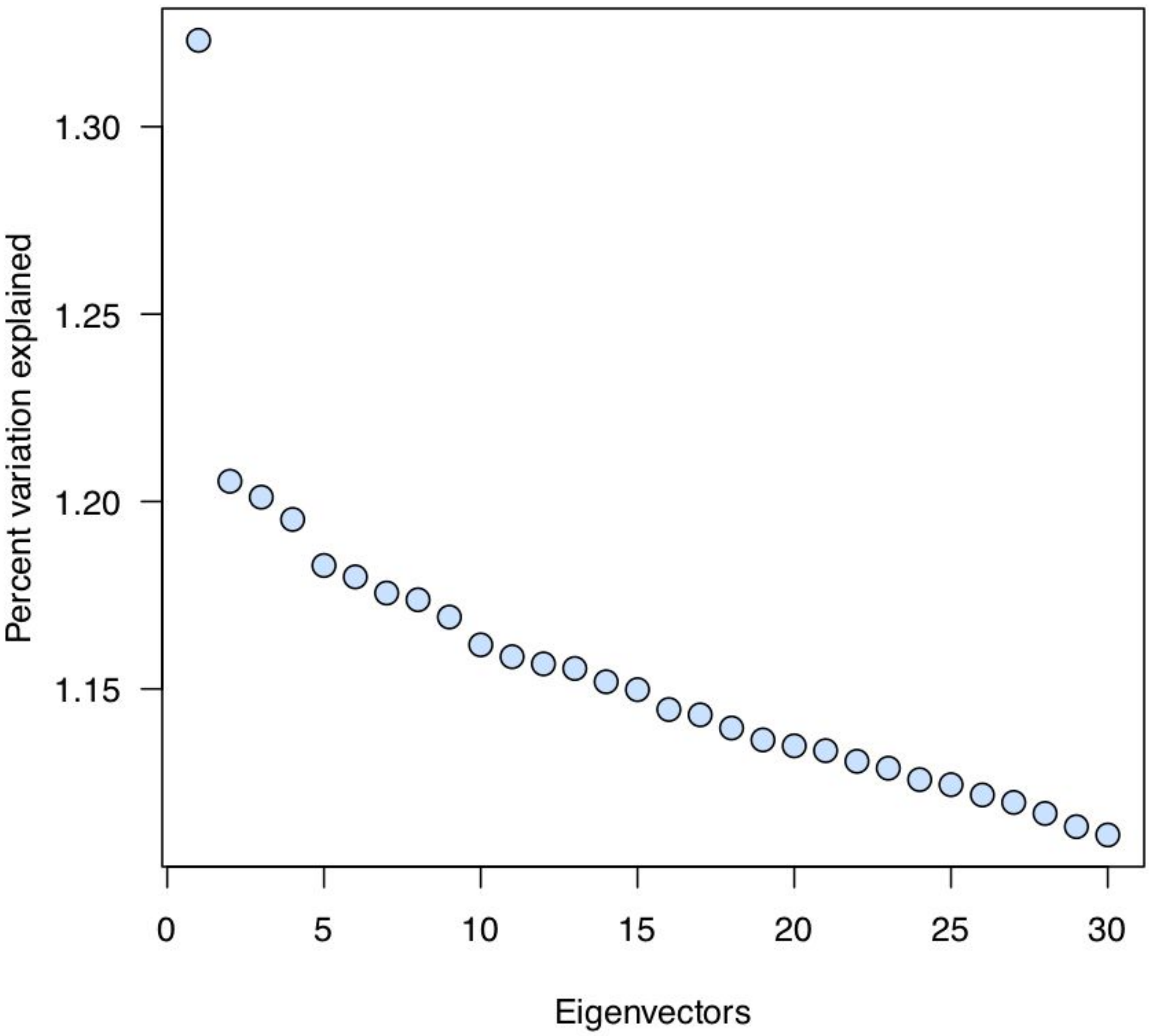


**Supplementary Figure 3.** Percent variation explained by the first 30 eigenvectors from an eigen-decomposition of a kinship matrix calculated using the SNP data.

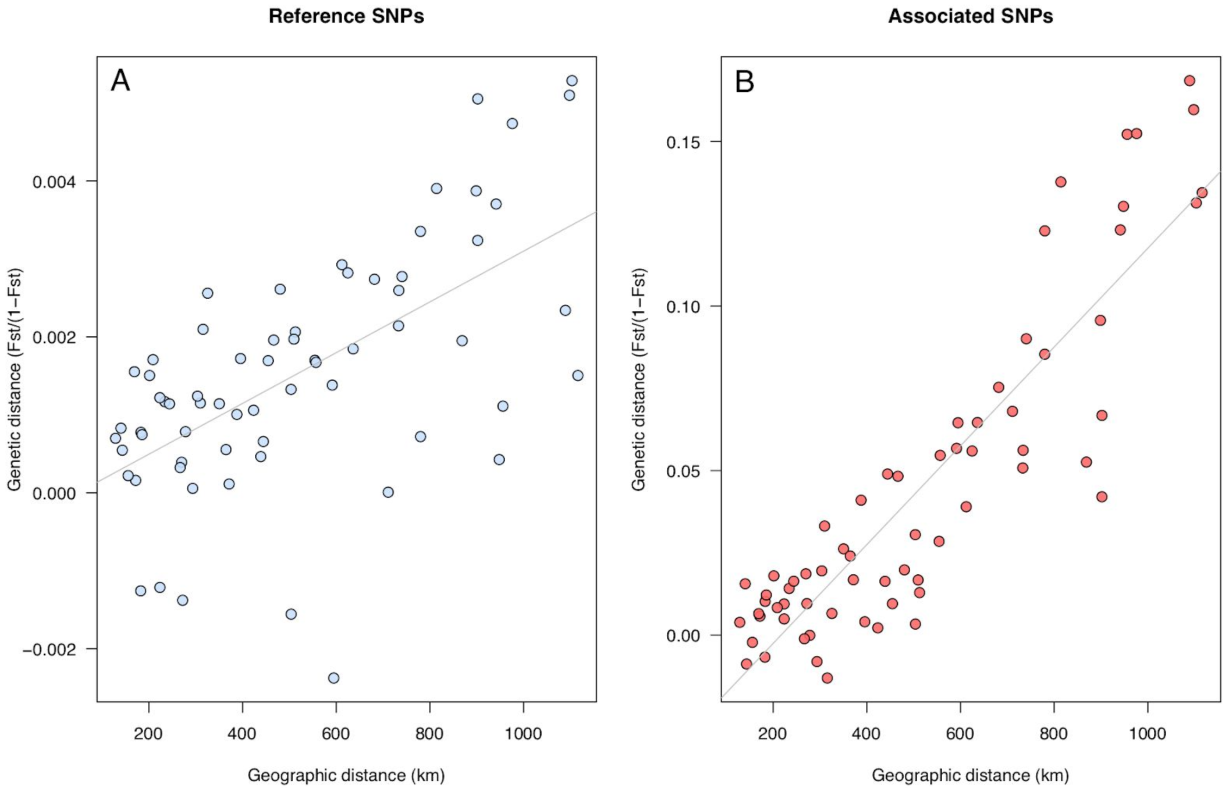


**Supplementary Figure 4**. Isolation by distance for A) 100,000 randomly selected SNPs (*r*=0.608, *p*<0.001) B) 111 climate-associated SNPs (reduced from 1,080 SNPs through LD clumping, *r*=0.894, *p*<0.001).

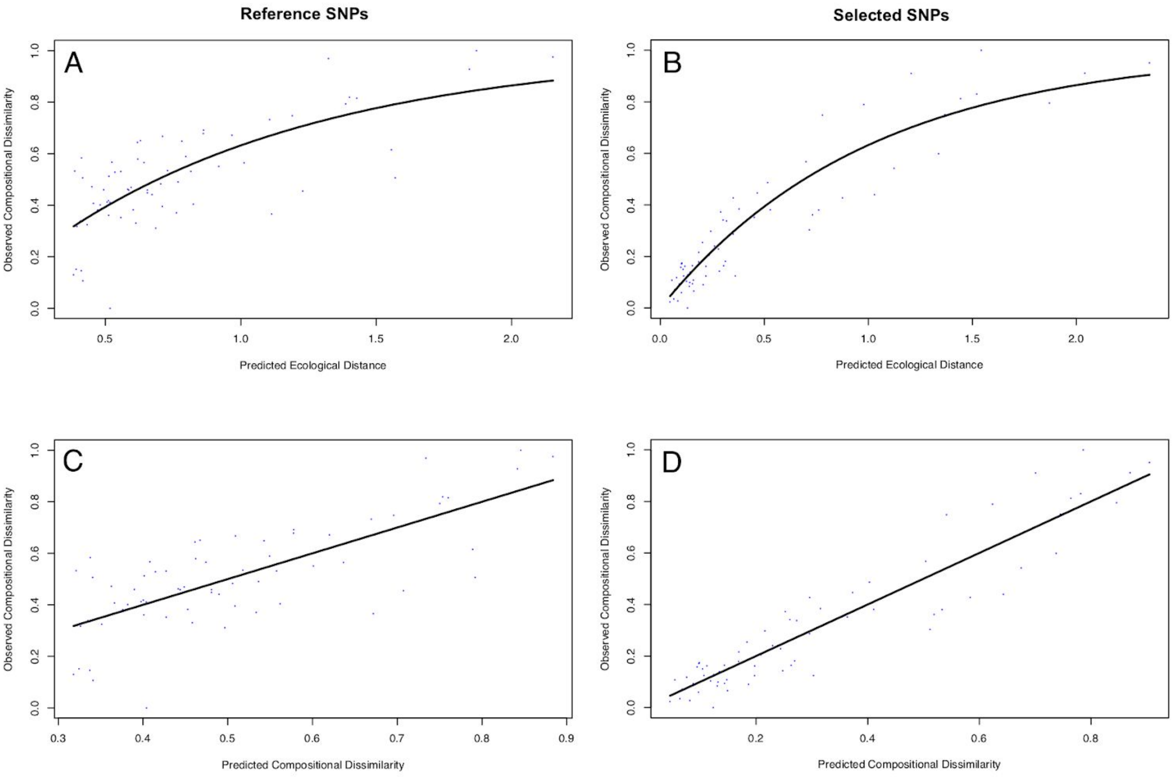


**Supplementary Figure 5.** Results from the generalized dissimilarity modelling (GDM) for 'reference' SNPs (A and C) or 'selected' SNPs (B and D). A and B show the predicted non-linear relationship from the GDM between environmental distance and genomic distance, with points indicating observed distances between pairs of populations. C and D display the relationship between predicted genomic distance (line) and observed genetic distances between pairs of populations (points).

**Supplementary Table 1.** Gene models associated with environmental variables

| ***P. tremula* gene name** | **Scaffold** | **Position** | **Arabidopsis homolog** | **Chromsome** | **Position** |
|---|---|---|---|---|---|
| *Potra008949g26261* | Potra008949 | 6079 - 7877 | AT4G17670.1 | 4 | 9833661 - 9834727 |
| *Potra002821g20059* | Potra002821 | 7631 - 12482 | AT3G60800.1 | 3 | 22467173 - 22469592 |
| *Potra001886g15018* | Potra001886 | 8377 - 11385 | AT2G29130.1 | 2 | 12524889 - 12527747 |
| *Potra002139g16570* | Potra002139 | 82262 - 84199 | AT1G69160.1 | 1 | 26000214 - 26001588 |
| *Potra000632g04828* | Potra000632 | 67370 - 71443 | AT2G42590.3 | 2 | 17731855 - 17733967 |
| *Potra001409g11957* | Potra001409 | 73866 - 83814 | AT5G37020.1 | 5 | 14630028 - 14634387 |
